# Hippo signaling-linked circulating microRNA networks are associated with cardiovascular disease in type 2 diabetes

**DOI:** 10.1210/jendso/bvag229

**Published:** 2026-10-14

**Authors:** Zein Mersini Besharat, Sofia Trocchianesi, Giuseppina Emanuela Grieco, Tanja Milena Autilio, Caterina Alfano, Angela Dardano, Daniela Fignani, Elena Splendiani, Anna Citarella, Claudia Sabato, Zaira Spinello, Giuseppina Catanzaro, Agnese Po, Manuela Petti, Lorenzo Farina, Mary Anna Venneri, Davide Lauro, Gian Paolo Fadini, Francesco Dotta, Guido Sebastiani, Andrea Natali, Elisabetta Ferretti

**Affiliations:** Department of Experimental Medicine, Sapienza University of Rome, Rome 00161, Italy; Sylvester Comprehensive Cancer Center, University of Miami, Miami, FL 33136, USA; Department of Public Health Sciences, University of Miami, Miami, FL 33136, USA; Department of Experimental Medicine, Sapienza University of Rome, Rome 00161, Italy; Max Delbrück Center–Berlin Institute for Medical Systems Biology (MDC-BIMSB), Berlin 10115, Germany; Department of Medicine, Surgery and Neuroscience, University of Siena, Siena 53100, Italy; Department of Experimental Medicine, Sapienza University of Rome, Rome 00161, Italy; Department of Computer, Control and Management Engineering, Sapienza University of Rome, Rome 00161, Italy; Department of Clinical and Experimental Medicine, University of Pisa, Pisa 56126, Italy; Department of Medicine, Surgery and Neuroscience, University of Siena, Siena 53100, Italy; Department of Experimental Medicine, Sapienza University of Rome, Rome 00161, Italy; Department of Life, Health and Health Professions Sciences, Link Campus University, Rome 00165, Italy; Laboratory of Preclinical and Translational Research, IRCCS CROB Centro di Riferimento Oncologico della Basilicata, Rionero in Vulture, PZ 85028, Italy; Department of Life, Health and Health Professions Sciences, Link Campus University, Rome 00165, Italy; Department of Life, Health and Health Professions Sciences, Link Campus University, Rome 00165, Italy; Department of Radiological, Oncological & Pathological Sciences, Sapienza University of Rome, Rome 00161, Italy; Department of Computer, Control and Management Engineering, Sapienza University of Rome, Rome 00161, Italy; Department of Computer, Control and Management Engineering, Sapienza University of Rome, Rome 00161, Italy; Department of Experimental Medicine, Sapienza University of Rome, Rome 00161, Italy; Department of Systems Medicine, Tor Vergata University, Rome 00133, Italy; Department of Medicine, University of Padova, Padua 35121, Italy; Department of Medicine, Surgery and Neuroscience, University of Siena, Siena 53100, Italy; Department of Biomedical Science, Consiglio Nazionale delle Ricerche, Rome 00185, Italy; Department of Medicine, Surgery and Neuroscience, University of Siena, Siena 53100, Italy; Department of Clinical and Experimental Medicine, University of Pisa, Pisa 56126, Italy; Department of Systems Medicine, Tor Vergata University, Rome 00133, Italy

**Keywords:** circulating miRNA, type 2 diabetes, cardiovascular disease, coexpression network, Hippo signaling pathway

## Abstract

**Context:**

Type 2 diabetes (T2D) markedly increases the risk of cardiovascular disease (CVD). Circulating microRNAs (miRNAs) are promising biomarkers for patient stratification and may provide mechanistic insight into dysregulated pathways in T2D-associated CVD.

**Objective:**

This work aimed to identify circulating miRNA signatures in T2D patients with CVD (T2D-CVD) vs T2D patients without CVD using a novel network-based medicine approach.

**Methods:**

Patients (n = 110) were stratified into T2D and T2D-CVD groups based on the absence or presence of macrovascular complications. Circulating miRNAs, proinflammatory cytokines, and growth factors were profiled in a discovery cohort (n = 80) and confirmed in a validation cohort (n = 30). Differential expression, network-based approaches, and pathway enrichment analyses identified miRNA signatures, coexpression communities, hub miRNAs, and associated signaling pathways.

**Results:**

T2D-CVD patients showed a set of elevated inflammatory cytokines and circulating miRNAs. Particularly, 19 miRNAs were differentially expressed between T2D-CVD and T2D patients. Pathway enrichment of these miRNAs highlighted Hippo signaling together with transforming growth factor-β, mechanistic target of rapamycin, Wnt, and PI3K-Akt pathways. Network analysis identified a coexpression community of 34 miRNAs, including hub miRNAs let-7a-5p, miR-132-3p, and miR-1306-3p, all linked to Hippo signaling. Key miRNA interactions were validated, and differentially expressed and hub miRNAs both correlated with inflammatory mediators such as PAR-1, CXCL1, and NEMO, which are linked to Hippo pathway regulation.

**Conclusion:**

This study identified a network of circulating miRNAs associated with inflammatory cytokines and Hippo signaling pathway in T2D-CVD. These findings reveal novel molecular aspects of the systemic inflammation characterizing T2D-CVD and support the potential of Hippo-related circulating miRNAs as mechanistic biomarkers for cardiovascular risk in T2D.

The prevalence of type 2 diabetes mellitus (T2D) continues to rise globally, substantially affecting patients' quality of life and increasing cardiovascular disease (CVD) risk and mortality. CVD accounts for more than half of deaths in T2D, and current risk scores incompletely capture the heterogeneity in CV outcomes, underscoring the need for mechanistic biomarkers to refine risk stratification and guide precision medicine approaches (1-5).

Circulating microRNAs (miRNAs) are small noncoding RNAs released into the bloodstream that regulate gene expression in multiple tissues and can be measured with minimally invasive procedures. They are remarkably stable in plasma and have been linked to key pathogenic processes in T2D, including insulin resistance, β-cell dysfunction, vascular remodeling, and inflammation (6, 7).

One major challenge is that circulating miRNAs reflect complex, multicellular communication networks that cannot be fully captured by conventional analyses. Network medicine offers a framework to address this complexity by integrating large-scale molecular data into interaction networks, where highly connected nodes and communities can reveal disease-relevant regulatory modules.

In the specific setting of T2D and its CV complications, network medicine allows the detection of disease-relevant subnetworks that highlight shared or distinct molecular mechanisms, thereby supporting the identification of mechanistic biomarkers (8).

In this study, we combined high-throughput profiling of circulating miRNAs and inflammatory molecules with network and pathway analyses in T2D patients with and without CVD. Our primary aim was to identify miRNA signatures and hub miRNAs that characterize T2D patients with CV complications and to link these signatures to specific signaling pathways, with a focus on Hippo signaling. Indeed, the Hippo signaling pathway is emerging as a key integrator of metabolic stress, inflammation, and tissue remodeling both in pancreatic and cardiovascular biology (9, 10). Yet, the relationship between circulating miRNAs, Hippo signaling, and the immune-inflammatory milieu in T2D patients with CVD has not been sufficiently characterized.

By integrating coexpression communities, differential network analysis, and targeted validation, we delineate a miRNA regulatory axis that connects systemic inflammation and Hippo pathway activity to CV risk in T2D, with potential implications for pathophysiological biomarker development and precision medicine.

## Materials and methods

### Study population

This was an observational, single-center, cross-sectional case-control study of prevalent CVD. Eligible patients with T2D (n = 110) were consecutively recruited during routine visits to the outpatient diabetes clinic at the University of Pisa and classified, at the time of blood sampling, as T2D without clinically manifest CVD (n = 55) or T2D with documented macrovascular disease (n = 55), defined by a history of myocardial infarction, stroke, or lower-extremity arterial disease. “Consecutive” refers to the recruitment method; group assignment was determined by prevalent CVD status and was not randomized. Patients were consecutively recruited at the University of Pisa based on prespecified inclusion criteria. Patients' characteristics are reported in Table 1 and Supplementary Table S1. The study was approved by the local ethical review board (approval ref. 5943). Participants were recruited in the outpatient setting, and all enrolled participants personally provided written informed consent before any study procedures. A previous stroke was identified from documented medical history and did not represent recruitment during an acute stroke. Individuals unable to understand the study information, communicate a voluntary decision, or personally provide written informed consent were not enrolled; surrogate consent was not used.

**Table 1:** Medication, lifestyle, and clinical characteristics of the study cohorts

Characteristic	Discovery cohort (n = 80)			Validation cohort (n = 30)			Discovery + Validation cohort (n = 50)		
	T2D-CVD (n = 40)	T2D (n = 40)	*P*	T2D-CVD (n = 15)	T2D (n = 15)	*P*	T2D-CVD (n = 25)	T2D (n = 25)	*P*
**Demographics**									
Age, y	63.7 (5.4)	63.1 (8.3)	.693	65.4 (8.6)	65.7 (6.1)	.904	65.1 (7.3)	66.5 (7.3)	.651
BMI	28.7 (3.8)	29.6 (4.8)	.373	29.1 (5.2)	28.4 (4.9)	.730	28.9 (3.8)	29.9 (4.8)	.424
Sex, male/female (n)	32/8	28/12	.302	11/4	8/7	.253	18/7	13/12	.143
**Lipid profile**									
Total cholesterol, mmol/L	4.00 (0.86)	5.01 (0.97)	<.001	3.90 (0.73)	4.80 (0.76)	.005	3.93 (0.88)	4.86 (0.75)	<.001
LDL cholesterol, mmol/L	2.27 (0.70)	3.16 (0.81)	<.001	2.11 (0.52)	2.94 (0.83)	.004	2.20 (0.70)	2.99 (0.74)	<.001
Apolipoprotein B, mg/dL	75 (20)	96 (23)	<.001	71 (16)	98 (23)	.016	71 (18)	91 (21)	<.001
Renal and cardiac biomarkers									
Albumin/creatinine ratio, mg/mmol	7.6 (22.1)	11.7 (44.8)	.628	2.6 (3.7)	3.3 (8.7)	.785	3.9 (6.8)	2.9 (7.1)	.611
B-type natriuretic peptide, pg/mL	57 (50)	38 (39)	.060	70 (59)	28 (31)	.021	65 (53)	38 (40)	.052
**Smoking**									
Smokers, current/ex (n)	7/28	1/15	—	3/8	2/4	.171	5/22	1/11	<.001
Pack-years	46 (29)	31 (28)	.086	59 (47)	49 (16)	.543	59 (41)	38 (32)	.181
**Medication (n)**									
β-Blockers	26	8	<.001	9	1	.001	19	4	<.001
Statins	38	10	<.001	14	4	<.001	23	6	<.001
Insulin	10	2	.025	5	1	.025	7	2	.064
Calcium channel blockers	15	6	.042	4	0	.013	10	4	.064
NSAIDs	34	9	<.001	9	3	.002	25	7	<.001

Data are presented as mean (SD) for continuous variables and as number of patients (n) for categorical variables, unless otherwise indicated. *P* values refer to the comparison between the T2D-CVD and T2D groups within each cohort.

Continuous variables were compared using the Mann-Whitney *U* test; categorical variables using the Fisher exact test.

Abbreviations: ACR, albumin/creatinine ratio; BMI, body mass index; BNP, B-type natriuretic peptide; CVD, cardiovascular disease; LDL, low-density lipoprotein; NSAIDs, nonsteroidal anti-inflammatory drugs; T2D, type 2 diabetes; T2D-CVD, type 2 diabetes with cardiovascular disease.

### Plasma samples collection and processing

Plasma-EDTA blood was collected and processed following standard operating procedures to obtain plasma. Briefly, blood samples were collected in K2-EDTA tubes, inverted 10 times, and stored upright at room temperature (15-25 °C), and processed within 2 hours from blood draw. An initial centrifuge was performed at 1800*g* for 10 minutes at 15 to 25 °C to separate blood cells from plasma. Then, plasma was collected in smaller tubes (ie, 2 mL—sterile, apyrogen- and nuclease-free) avoiding touching the white blood cells interphase (leaving 2-3 mm of plasma layer over the leukocytes) and further centrifuged at 1200*g* for 20 minutes at 10 °C to remove contaminant cells and platelets (11).

### Study design

We conducted a cross-sectional case-control study integrating high-throughput profiling of circulating small RNAs and inflammatory molecules with network-based analyses.

Small RNA sequencing was performed in the discovery cohort to obtain circulating miRNA profiles. For this initial discovery analyses, we selected 40 T2D and 40 T2D-CVD patients (see Table 1). We then applied differential expression (DE) analysis, coexpression network and community detection, and differential network analysis to identify disease-associated miRNA signatures and hubs. Pathway enrichment analyses of validated miRNA targets and correlation analyses with clinical and inflammatory readouts were used to infer functional pathways.

Selected miRNAs and miRNA subnetworks were technically validated by droplet digital polymerase chain reaction (ddPCR) (Fig. 1) in an independent validation cohort including 15 T2D and 15 T2D-CVD patients (see Supplementary Table S1) (12). For validation of network hubs, we used plasma from 25 T2D and 25 T2D-CVD patients (15 from the validation cohort plus 10 from the discovery cohort in each group, the latter selected at random among discovery-cohort patients with sufficient residual plasma/RNA available and with no selection based on miRNA expression values, to avoid circularity/selection bias) (see Table 1 and Supplementary Table S1) (12).

**Figure 1:**
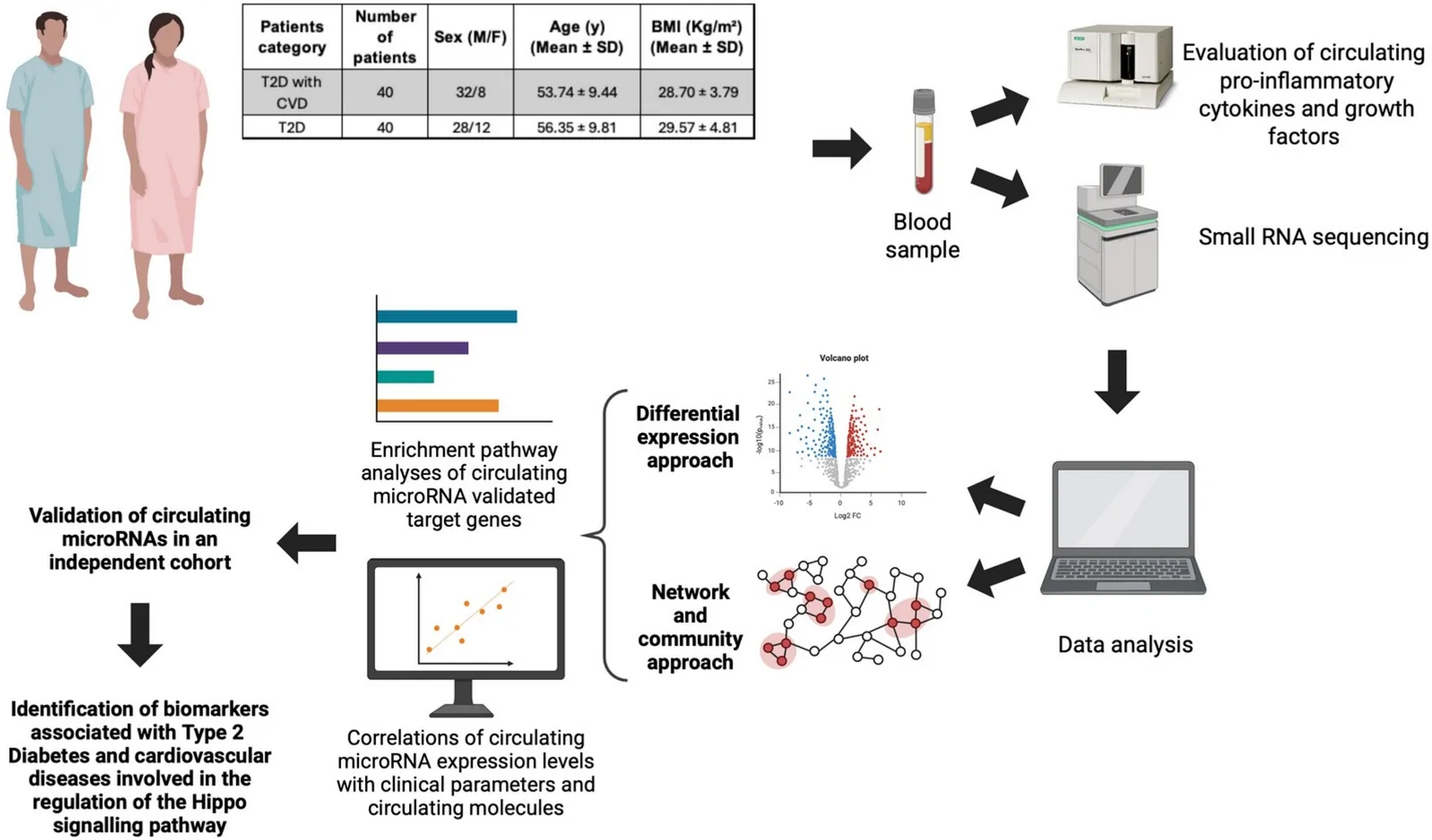
Study design. Type 2 diabetes (T2D) patients without cardiovascular disease (CVD) (T2D) and with CVD (T2D-CVD) were included in the study, and blood samples were collected for circulating proinflammatory cytokines and growth factors evaluation and small RNA sequencing. Circulating proinflammatory cytokines and growth factors were evaluated. Circulating microRNA (miRNA) profiles were obtained and differential expression analysis along with network and community analyses were applied to identify miRNAs associated with T2D-CVD. Enrichment pathway analyses of validated target genes of the circulating miRNAs and correlation of miRNAs with clinical parameters and circulating molecules were performed. Circulating miRNAs were validated in a second cohort (n = 30) and biomarkers associated with T2D-CVD involved in the regulation of the Hippo signaling pathway were identified. Created in BioRender under license. Autilio, T.M. (2026). https://BioRender.com/di5reom.

### Small RNA sequencing

Total RNA was extracted from 200 μL of plasma using the Norgen RNA extraction kit (catalog No. 55000), and small RNA libraries were prepared with the QIAseq miRNA library kit (catalog No. 331505, Qiagen) (11). QIASeq miRNA library chemistry assigns a unique molecular index during the reverse transcription step to every mature miRNA molecule, to enable unbiased and accurate miRNome-wide quantification of mature miRNAs through next-generation sequencing. Then, libraries quality control was performed quantifying their concentration through QUBIT 3.0 spectrofluorometer (Qubit dsDNA HS Assay Kit, catalog No. Q32854, Thermofisher Scientific) and assessing their length using capillary electrophoresis in Bioanalyzer 2100 (Agilent High Sensitivity DNA kit catalog No. 5067-4626, Thermofisher Scientific). Only libraries showing a peak between 175 and 185 bp were included. Normalized libraries were pooled and sequenced on an Illumina NovaSeq 6000 platform using 75-bp single-end reads (NovaSeq 6000 SP Reagent Kit [100 cycles] (catalog No. 20027464], NovaSeq XP 2-Lane Kit cat. 20021664, Illumina) using the XP protocol applying 75 × 1 single reads.

### Circulating inflammatory mediators

Circulating proinflammatory cytokines and growth factors were evaluated in the same plasma samples and differences between T2D and T2D-CVD patients were assessed using either the *t* test or Mann-Whitney *U* test, as appropriate. Box plots reporting expression levels of molecules and clinical parameters in T2D and T2D-CVD were generated in R (ggplot2 package). In detail, all biomarkers were analyzed by the proximity extension assay technique using the Proseek Multiplex CVD 96 × 96 reagents kit (Olink Bioscience) at the Clinical Biomarkers Facility, Science for Life Laboratory, Uppsala. Oligonucleotide-labeled antibody probe pairs could bind to their respective targets present in the plasma sample and the addition of a DNA polymerase led to an extension and joining of the two oligonucleotides and formation of a PCR template. Universal primers were used to preamplify the DNA templates in parallel. Finally, the individual DNA sequences were detected and quantified using specific primers by microfluidic real-time quantitative PCR chip (96.96, Dynamic Array IFC, Fluidigm Biomark). The chip was run with a Biomark HD instrument. All samples were analyzed in the same run. Data analysis was performed by a preprocessing normalization procedure using Olink Wizard for GenEx (Multid Analyses). All data are presented as arbitrary units. Technical information about the assays is available on the Olink homepage (http://www.olink.com).

### Small RNA-sequencing, network, and pathway analyses

#### MicroRNA-sequencing data analyses

Raw small RNA counts were extracted from FASTQ files with the sRNAbench tool from sRNAtoolbox suite (sRNAbench and sRNAtoolbox 2019: intuitive fast small RNA profiling and DE-sRNA bench bioinformatic pipeline (https://arn.ugr.es/srnatoolbox/srnabench/), preprocessed with an ad hoc script in R, and normalized with median of ratios method with DESeq2 R package (moderated estimation of fold change [FC] and dispersion for RNA-sequencing [RNA-seq] data with DESeq2) prior to perform DE analysis.

Raw FASTQ files were uploaded to the sRNAbench webserver (batch mode) selecting Human (GRCh38_p10) as species, Qiagen (with unique molecular index) as reads preprocessing, and leaving all other parameters to their default value.

Small RNAs with at least 2 reads in all samples were kept and small RNA datasets with fewer than 2 features or in which every feature contained at least 1 zero were removed. DE miRNAs were identified using DESeq2 and miRNAs with log2 FC greater than or equal to 0.6, false discovery rate (FDR) less than 0.05 were considered statistically significant. Heatmap and volcano plots were generated in R using pheatmap and ggplot2 packages.

#### Network and community analyses

Coexpression networks for T2D-CVD and T2D respectively were constructed by computing the Spearman rank correlation coefficient to evaluate the level of coexpression among miRNAs and by selecting (±)0.7 as a threshold. On these networks the weighted Louvain algorithm was applied to identify communities of highly coexpressed miRNAs. Finally, the two coexpression networks were compared through the construction of a differential coexpression network, “T2D-CVD vs T2D.” An miRNA differential coexpression network is a network in which a link between two miRNAs represents a statistical change in the coexpression of the two miRNAs across the considered cohorts. The edge weight z can be either positive or negative and its absolute value quantifies the magnitude of the statistical change. To compute each z we used the following formula:


(1) $$Z=\frac{z_{T2D-CVD}-\;z_{T2D}}{\sqrt{\frac{1}{n_{T2D-CVD}-3}\;+\;\frac{1}{n_{T2D}-3}\;\;}}$$

where $z_{T2D+\mathrm{CVD}}$ and $z_{T2D}$ are the Fisher *Z* transformation of, respectively, the correlation between the miRNAs in the T2D-CVD and T2D cohorts, while $n_{T2D-CVD}$ and $n_{T2D}$ are the number of samples in the cohorts. Positive *z* scores represent increases of coexpression in the first cohort, while negative *z* scores represent increases of coexpression in the second network. We selected 3 as a threshold to focus on statistically significant changes. Hubs of the network were identified by selecting the nodes whose degree (number of connections) belongs to the fifth and 95th percentile of the network's degree distribution.

#### Pathway enrichment analyses

MiRNAs of interest were used as input and based on validated target genes of the miRNAs enrichment pathway analysis was performed using miRpath v.4 (13) (*P* < .05).

### Correlation analyses

Clinical variables and circulating molecules were compared between groups using χ^2^ tests for categorical variables and *t* tests or Mann-Whitney *U* tests for continuous variables; *P* values were adjusted for multiple testing using the FDR. Correlations between miRNAs and clinical or inflammatory parameters were assessed using the Spearman rank correlation for continuous variables and point-biserial correlation or one-way analysis of variance for categorical variables, as appropriate. Circos plots were generated with a custom Python script (Python 3.10; matplotlib 3.10.9, NumPy 2.2.6, pandas 2.3.3). For each group (T2D and T2D-CVD), Spearman rank correlations between the expression levels of the DE miRNAs and clinical variables/inflammatory factors were displayed. Only associations remaining statistically significant after Benjamini-Hochberg correction for multiple testing (FDR-adjusted *P* < .05) were shown.

### Droplet digital polymerase chain reaction validation of selected microRNAs

Sixteen miRNAs, the 3 hub miRNAs, and their networks, were validated in the independent validation cohort. Circulating miRNAs levels were quantified using ddPCR (Bio-Rad Laboratories). A reverse transcription was performed for each sample, starting from 2 μL of RNA using the miRCURY LNA RT Kit (catalog No. 339340, QIAGEN), following the manufacturer's protocol. Then, 8 μL of each diluted complementary DNA template were added to a ddPCR reaction mixture containing 11 μL of ddPCR Supermix for Probes (no dUTP) (catalog No. 1863025; Bio-Rad Laboratories), 1.1 μL of specific miRCURY LNA miRNA assay (catalog No. YP00205727 hsa-let-7a-5p; catalog No. YP00205711 hsa-let-7e-5p; catalog No. YP00204565 hsa-let-7g-5p; catalog No. YP02119520 hsa-miR-1306-3p; catalog No. YP00206035 hsa-miR-132-3p; catalog No. YP00205867 hsa-miR-148a-3p; catalog No. YP00204007 hsa-miR-151a-5p; catalog No. YP00204555 hsa-miR-196b-5p; catalog No. YP00204536 hsa-miR-199a-3p and hsa-miR-199b-3p; catalog No. YP00204482 hsa-miR-200c-3p; catalog No. YP00205624 hsa-miR-423-5p; catalog No. YP02102613 hsa-miR-4446-3p; catalog No. YP00205657 hsa-miR-505-5p; catalog No. YP02115612 hsa-miR-664a-5p; QIAGEN) and 1.9 μL nuclease-free water. For droplets generation, 20 μL of each ddPCR reaction mixture was loaded in disposable droplet generator cartridges (catalog No. 1864008; Bio-Rad Laboratories) along with 70 μL of droplet generation oil for probes (catalog No. 1863005; Bio-Rad Laboratories) and placed into a QX200TM Droplet Generator (Bio-Rad Laboratories). Generated droplets were transferred to 96-well PCR plates (catalog No. 12001925; Bio-Rad Laboratories). PCR was carried out in a C1000 Touch Thermal Cycler (Bio-Rad Laboratories) using the following thermal cycling program: 5 minutes at 95 °C for enzyme activation, followed by 40 cycles of 30 seconds at 95 °C and a 1-minute annealing/extension step at the appropriate temperature based on the primer/probe set, 5 minutes at 4 °C, 5 minutes at 90 °C for enzyme deactivation followed by infinite hold at 4 °C.

Finally, the QX200 Droplet Reader (Bio-Rad Laboratories) was used to read the fluorescence signals, and QuantaSoft software was used for ddPCR data analysis (version 1.7.4; Bio-Rad Laboratories).

## Results

### Analysis of the clinical characteristics of type 2 diabetes and type 2 diabetes–cardiovascular disease individuals

The discovery cohort included a total of n = 80 individuals subdivided into T2D patients with CVD (n = 40) and T2D without clinical signs of CVD (n = 40). Clinical characteristics of T2D with CVD (T2D-CVD) and T2D individuals are reported in Table 1 and Supplementary Table S1 (12).

Statistically significant differences were observed in several medication and lifestyle-related variables. Specifically, T2D-CVD patients were more frequently treated with β-blockers, statins, insulin, and calcium channel blockers, and had a longer smoking history (see Table 1 and Supplementary Fig. S1A) (12).

Despite the presence of CV comorbidities, T2D-CVD patients exhibited lower levels of total cholesterol, low-density lipoprotein cholesterol, and apolipoprotein B, likely due to intensive lipid-lowering therapies (Supplementary Fig. S1B) (12). Conversely, albumin-to-creatinine ratio, and B-type natriuretic peptide were elevated in T2D-CVD patients, reflecting renal and cardiac involvement (see Table 1).

### A set of circulating microRNAs is differentially expressed in plasma of type 2 diabetes–cardiovascular disease patients

Small RNA sequencing detected 271 circulating miRNAs across plasma samples (Supplementary Data File 1 (12) and Supplementary Fig. S2 (12)).

DE analysis identified 19 miRNAs that discriminated T2D-CVD from T2D, with 7 miRNAs upregulated (let-7f-5p, let-7a-5p, miR-200c-3p, miR-151a-5p, miR-98-5p, miR-4446-3p, miR-1294) and 12 miRNAs downregulated (miR-99a-5p, miR-136-3p, miR-323a-3p, miR-100-5p, miR-369-3p, miR-483-5p, miR-122-5p, miR-203a-3p, miR-194-5p, miR-34a-5p, miR-192-5p, miR-197-3p) in T2D-CVD (log2 FC| ≥ 0.6, FDR < 0.05, sex and hemolysis as confounding factors) (Fig. 2A and 2B and Table 2).

**Figure 2:**
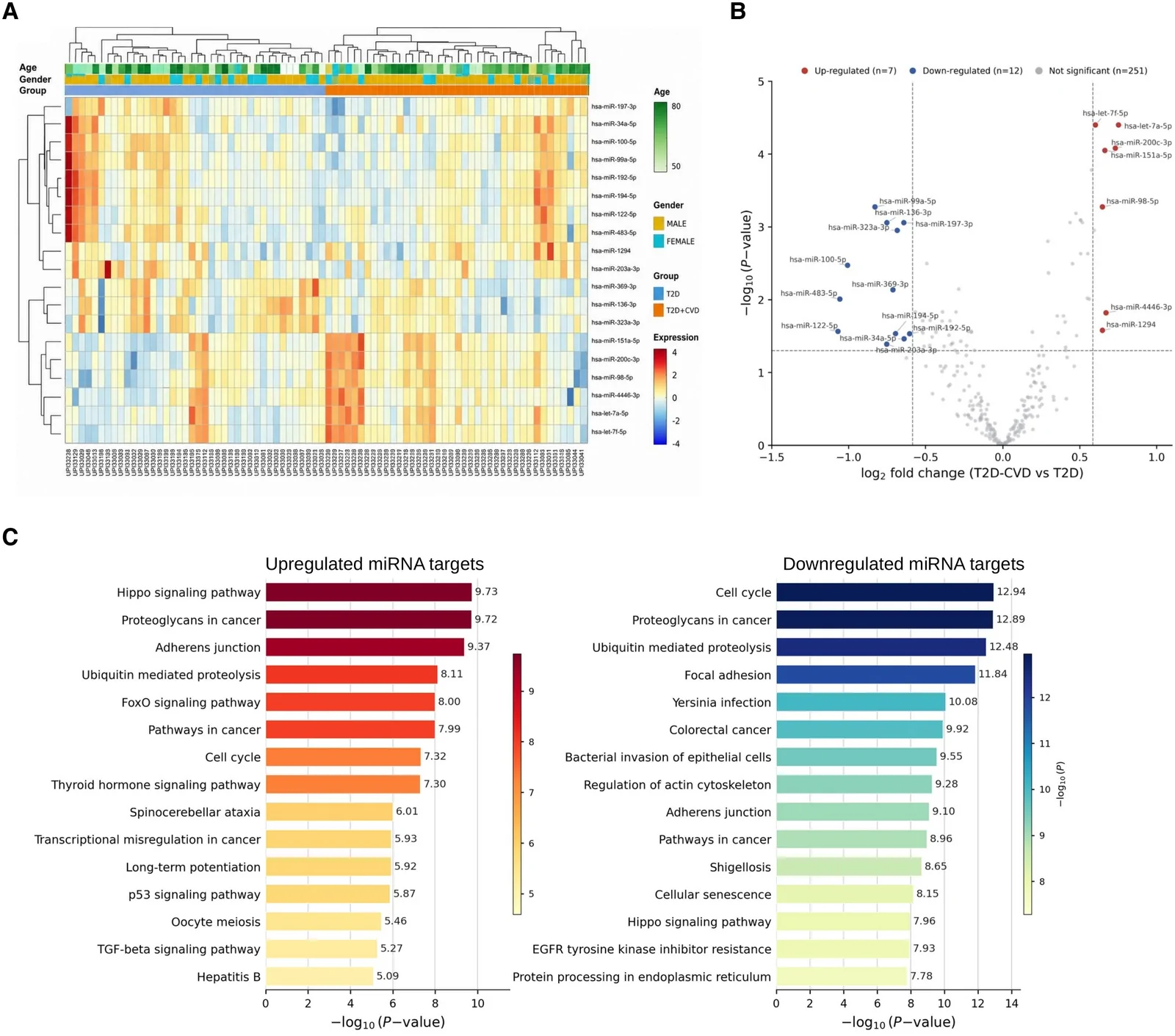
Circulating miRNA profiles in T2D and T2D-CVD patients. A, Unsupervised hierarchical clustering heatmap of the differentially expressed miRNAs across the 80 study participants (T2D, n = 40; T2D-CVD, n = 40). Rows represent individual miRNAs and columns individual participants; shading reflects row-scaled normalized expression (*z*-score; higher to lower relative expression), as indicated in the key. Top annotation bars denote age, sex, and clinical group; dendrograms show Euclidean distance/average-linkage clustering of miRNAs (rows) and participants (columns). B, Volcano plot of differential miRNA expression in T2D-CVD vs T2D. The x-axis shows the log_2_ fold change and the y-axis the −log_10_(*P*-value). Vertical and horizontal dashed lines indicate the significance thresholds (|log_2_ fold change| > 0.585, ie, fold change > 1.5, and *P*_adj_ < 0.05). Filled points denote significantly up-regulated (n = 7) and down-regulated (n = 13) miRNAs; open points are not significant (n = 251). Selected miRNAs are labelled. C, Top enriched pathways for the experimentally/predicted target genes of the up-regulated (left) and down-regulated (right) miRNAs. Bars represent enrichment significance as −log_10_(*P*-value); bar shading is graded according to the same −log_10_(*P*-value), as shown in the scales.

**Table 2:** Differentially expressed circulating microRNAs in type 2 diabetes with cardiovascular disease vs type 2 diabetes patients

Up in T2D-CVD vs T2D (n = 7)	Down in T2D-CVD vs T2D (n = 12)
hsa-let-7a-5p	hsa-miR-34a-5p
hsa-let-7f-5p	hsa-miR-99a-5p
hsa-miR-98-5p	hsa-miR-100-5p
hsa-miR-151a-5p	hsa-miR-122-5p
hsa-miR-200c-3p	hsa-miR-136-3p
hsa-miR-1294	hsa-miR-192-5p
hsa-miR-4446-3p	hsa-miR-194-5p
	hsa-miR-197-3p
	hsa-miR-203a-3p
	hsa-miR-323a-3p
	hsa-miR-369-3p
	hsa-miR-483-5p

The direction of change (up/down) refers to the expression of each miRNA in T2D-CVD patients relative to T2D patients. miRNAs were considered differentially expressed at log2 fold change greater than or equal to 0.6; false discovery rate less than 0.05.

Abbreviations: CVD, cardiovascular disease; miRNA, microRNA; T2D, type 2 diabetes; T2D-CVD, type 2 diabetes with cardiovascular disease.

Pathways enrichment analysis based on validated targets of these 19 miRNAs highlighted multiple signaling cascades relevant to cardiometabolic disease, including Hippo, transforming growth factor-β (TGF-β), mechanistic target of rapamycin (mTOR), Wnt, PI3K-Akt, glucagon, HIF-1, and mitogen-activated protein kinase (MAPK) pathways. These data support the concept that circulating miRNAs capture systemic molecular perturbations linking T2D to CV complications (Fig. 2C and Supplementary Table S2) (12).

### Coexpression network analysis defines a novel type 2 diabetes–cardiovascular disease–specific microRNA pattern

Coexpression network analysis revealed 6 distinct miRNA communities both in T2D and T2D-CVD. Most T2D-CVD communities overlapped with those in T2D; however, community 2 in T2D-CVD showed a unique composition, with more than half of its 34 miRNAs not represented in any T2D community (Fig. 3A-3C).

**Figure 3:**
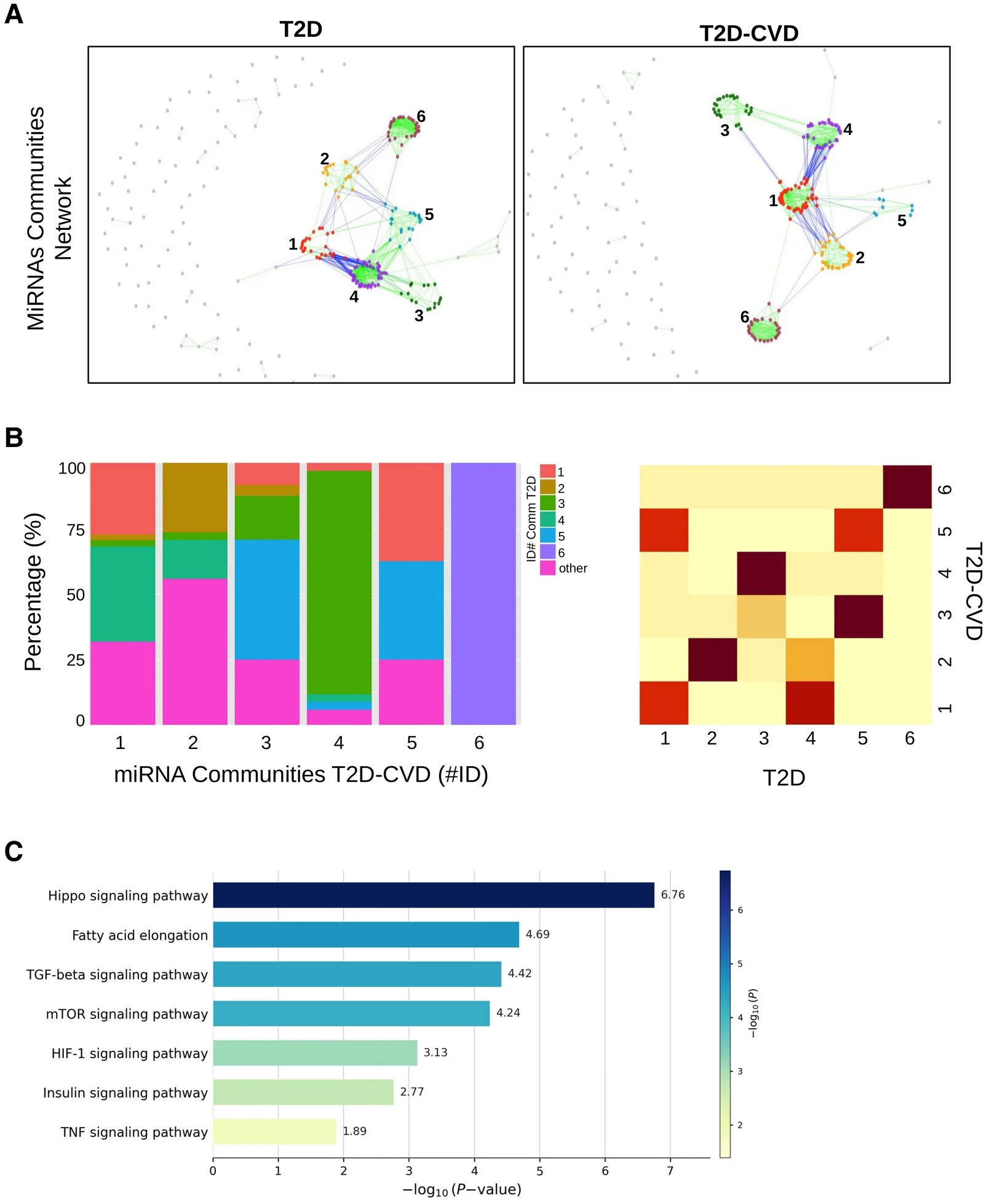
Coexpression network analysis reveals microRNA (miRNA) community and pathway insights in type 2 diabetes–cardiovascular disease (T2D-CVD) patients. A, Coexpression networks of circulating miRNAs in T2D (left) and T2D-CVD (right). Nodes represent miRNAs and edges their pairwise coexpression; densely connected modules (communities) identified by community-detection are highlighted in color and numbered, while miRNAs not assigned to a community are shown in gray. B, Correspondence between T2D-CVD and T2D miRNA communities. Left: stacked bar plot showing, for each T2D-CVD community (x-axis, ID), the percentage of its miRNAs assigned to each T2D community (color key; “other” denotes miRNAs unassigned to any T2D community). Right: heat map of the pairwise similarity between communities of the two cohorts, computed as the intersection over the size of the smaller set (darker = higher similarity). C, Top pathways enriched among the 34 T2D-CVD–specific community 2 miRNAs, ranked by decreasing statistical significance (only pathways with *P* < .05 are shown). Bars represent enrichment statistical significance as −log_10_(*P *value); bar color is graded by the same −log_10_(*P *value), as indicated in the color scale.

Notably, this T2D-CVD–specific community contained 6 of the 19 DE miRNAs (miR-1294, miR-122-5p, miR-192-5p, miR-194-5p, miR-197-3p, miR-483-5p) (see Fig. 3A-3C and Supplementary Table S3) (12).

Collectively, these findings delineate a distinct miRNA community uniquely associated with T2D-CVD.

To further investigate the potential role of plasma miRNAs in the molecular mechanisms underlying T2D-CVD, a pathway enrichment analysis was conducted using the 34 miRNAs (see Supplementary Table S3) (12) from the identified community. Pathway analysis of the 34 community-2 miRNAs indicated enrichment of mTOR, TGF-β, insulin, and Hippo signaling pathways, pointing to a coordinated regulatory module involved in metabolic stress, inflammation, and cardiovascular remodeling in T2D-CVD (Fig. 3C).

### Differential network analysis identifies Hippo-related hub microRNAs

Differential coexpression analysis comparing T2D-CVD and T2D networks identified subnetworks whose connectivity changed significantly between groups. In the positive differential network (stronger coexpression in T2D-CVD), 3 miRNAs emerged as hubs: let-7a-5p, miR-132-3p, and miR-1306-3p. Let-7a-5p was connected with a subnetwork of 7 miRNAs (let-7 g, let-7e, miR-151a-5p, miR-3615, miR-196b-5p, miR-200c-3p, miR-505), of which let-7a-5p, miR-151a-5p, and miR-200c-3p were also upregulated in T2D-CVD (Fig. 4A and 4B, left panel). Notably, let-7a-5p is upregulated in T2D-CVD compared to T2D, along with 2 other miRNAs (miR-151a-5p and miR-200c-3p), which are also upregulated in T2D-CVD. Pathway enrichment analysis of let-7a-5p and its 7 connected miRNAs revealed significant enrichment of relevant pathways of interest such as the Hippo signaling, TGF-β, extracellular matrix (ECM)-receptor interaction, MAPK, and arrhythmogenic right ventricular cardiomyopathy pathways (Supplementary Table S4) (12).

**Figure 4:**
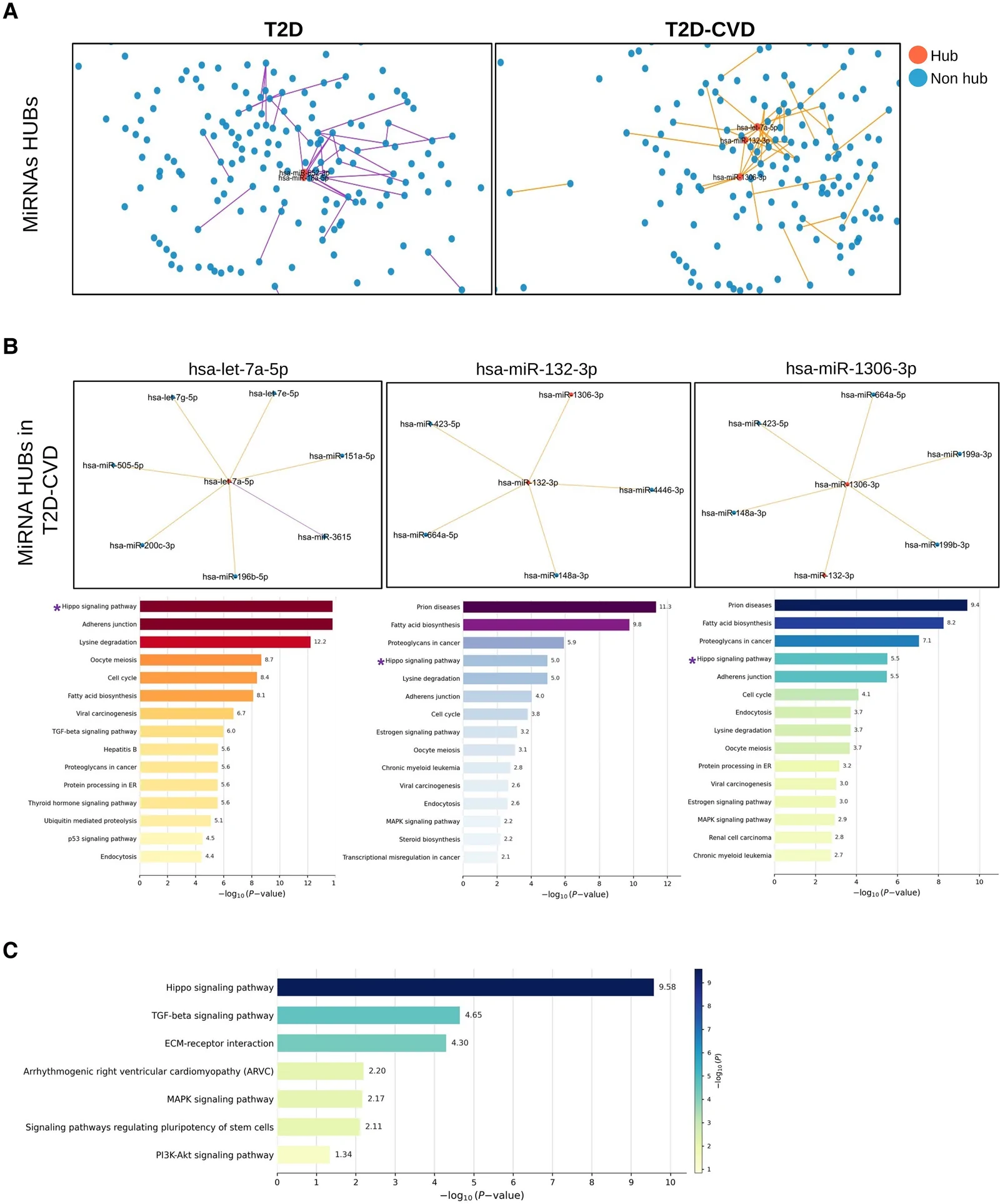
Differential miRNA network analysis reveals key hub miRNAs and Hippo pathway enrichment in T2D-CVD. A, Differential network analysis of co-expressed miRNAs in T2D-CVD and T2D patients. The negative component of the differential network (left panel, in purple) highlights the co-expression relationships which are significantly stronger in T2D patients. The positive component of the differential network (right panel, in orange) highlights the co-expression relationships which are significantly stronger in T2D-CVD patients. Three hub miRNAs are identified in the positive differential network: hsa-let-7a-5p, hsa-miR-132-3p and hsa-miR-1306-3p. B, Circulating hub miRNAs and their subnetworks in T2D-CVD patients: hub miRNA hsa-let-7a-5p connected with 7 miRNAs (left panel); hub miRNA hsa-miR-132-3p connected with 5 miRNAs (middle panel) and hub miRNA hsa-miR-1306-3p connected with 6 miRNAs (right panel). Purple lines represent co-expression relationships which are significantly stronger in T2D patients, while orange lines represent co-expression relationships which are significantly stronger in T2D-CVD patients. Top pathways enriched by miRNA hub network target genes are reported below each network; bars represent enrichment significance as −log_10_(*P*-value); bar shading is graded by the same −log_10_(*P*-value). C, Histogram of enriched pathways of the 3 hub circulating miRNAs in T2D-CVD, enriched pathways reported in order of decreasing *P*-value, *P* < .05. Bars represent enrichment significance as −log_10_(*P*-value); bar shading is graded by the same −log_10_(*P*-value).

Interestingly, miR-132-3p formed a subnetwork with 5 miRNAs including miR-1306-3p and miR-4446-3p, the latter significantly upregulated in T2D-CVD (see Fig. 4B).

Pathway enrichment of targets of these 3 hub networks consistently pointed to Hippo signaling, TGF-β signaling, ECM-receptor interaction, adrenergic signaling in cardiomyocytes, and arrhythmogenic right ventricular cardiomyopathy pathways. These findings identify let-7a-5p, miR-132-3p, and miR-1306-3p as central regulators within Hippo-associated miRNA circuits in T2D-CVD (see Fig. 4B, middle and right panels, Supplementary Table S4, and Fig. 4C) (12).

In the ddPCR validation cohort (25 T2D and 25 T2D-CVD), we confirmed the association of hub let-7a-5p with miR-505-5p and let-7e-5p, and hub miR-132-3p with miR-664a-5p, while miR-148a-3p, miR-423-5p, and miR-4446-3p showed a trend. These connections reproduced the differential coexpression patterns observed in the discovery cohort, although not all edges reached statistical significance. In contrast, no robust network connections were validated for hub miR-1306-3p, suggesting that its role may depend on context or be more sensitive to sample size (see Supplementary Table S1 and Fig. 5) (12). Overall, validated hub networks remained significantly enriched for the Hippo signaling pathway, which plays a critical role in modulating several pathways involved in inflammation and tissue regeneration in CVD (14-16).

**Figure 5:**
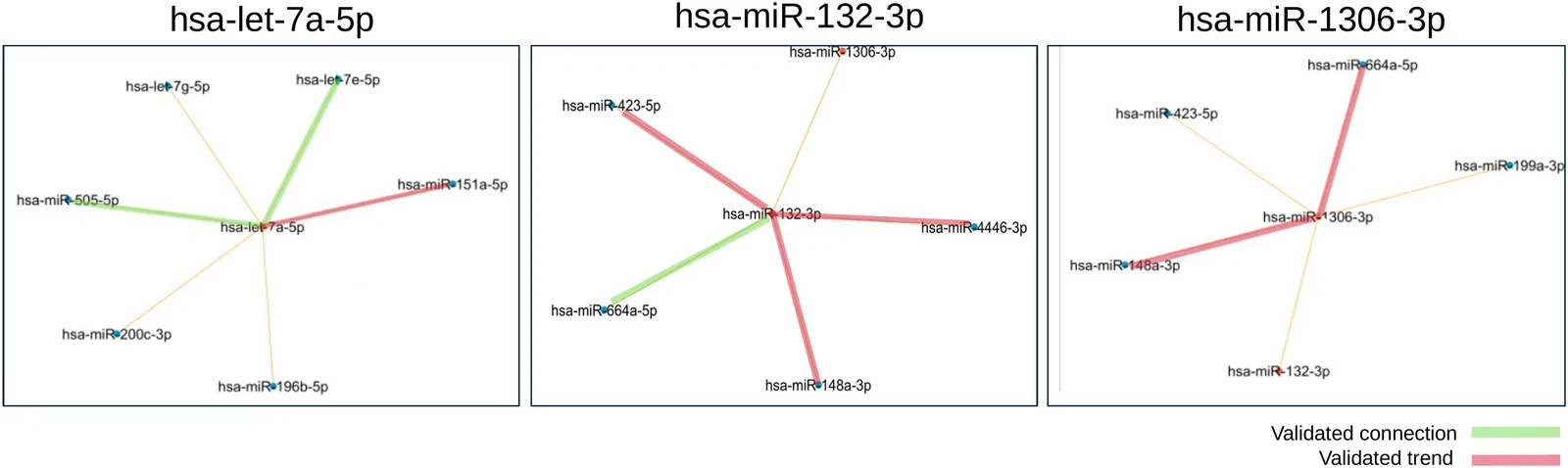
Validation of differential networks and their hub miRNAs in T2D-CVD. Validation of circulating hub miRNAs and their network miRNAs in T2D-CVD through ddPCR in a validation cohort of 25 T2D-CVD and 25 T2D patients (Supplementary Table S1 (12)). Orange lines represent co-expression relationships which are significantly stronger in T2D-CVD patients. Connections labelled “Validated connection” in the differential subnetwork plots were validated in the independent cohort (*P*-value < .05); connections labelled “Validated trend” did not reach statistical significance but showed a similar trend (*P*-value > .05); connections without any label were not validated.

### Inflammatory profile and integration with microRNA networks

To gain further insight into miRNAs molecular networks and immune-inflammatory alterations that might be connected to vascular alterations and remodeling, a comprehensive profiling of inflammatory cytokines/chemokines, soluble cytokine receptors, and molecules involved in atherosclerosis were evaluated in the same sample set.

Plasma profiling revealed that several inflammatory and CV biomarkers, including TRAIL, KLK6, CTSL1, MMP-12, NT-proBNP, CSTB, and AGRP, differed between T2D-CVD and T2D. Among these, KLK6, MMP-12, and NT-proBNP have been linked to Hippo pathway activity via regulation of YAP/TAZ. NT-proBNP, in particular, is an established biomarker of cardiac stress and adverse cardiovascular outcomes in T2D (Supplementary Table S5) (12). Notably, KLK6, MMP-12, and NT-pro.BNP have been described to be associated with the Hippo pathway, specifically via regulation or feedback with the YAP/TAZ transcriptional machinery (17-19). Moreover, NT-pro.BNP has been reported as a biomarker for cardiac stress, heart failure, and CV risk stratification in T2D patients with CVD (20). Together, these findings indicate a complex molecular and clinical phenotype in T2D-CVD patients, marked by altered inflammatory signaling and CV stress biomarkers, many of which converge on pathways such as Hippo signaling, known to mediate metabolic and vascular remodeling processes (21).

Furthermore, to explore the interplay between inflammatory mediators and circulating miRNAs, we performed correlation analyses.

A negative correlation of 6 miRNAs among the 19 DE miRNAs was observed with CXCL1 and NEMO for T2D patients (Fig. 6A, left panel). A positive correlation of 2 miRNAs and a negative correlation of 2 miRNAs among the 19 DE miRNAs was observed with PAR-1 and NEMO in T2D-CVD patients (see Fig. 6A, right panel). Importantly, NEMO, PAR-1, and CXCL1 are upstream or downstream regulators of the Hippo-signaling pathway. NEMO phosphorylates YAP at Ser128 blocking 14-3-3 binding and promoting nuclear localization, placing NEMO upstream of YAP activation (22). PAR-1 activates YAP/TAZ by inhibiting Lats1/2 kinase via G(12/13) and Rho GTPase, positioning PAR-1 as an upstream input to Hippo signaling (23), whereas CXCL1 expression is induced by MST1 through Hippo signaling (24). Thus, even though PAR-1, CXCL1, and NEMO were not among the molecules whose expression levels showed statistically significant differences in T2D and T2D-CVD patients (Fig. 6B), we proceeded to explore their possible association with community 2 and hub miRNAs.

**Figure 6:**
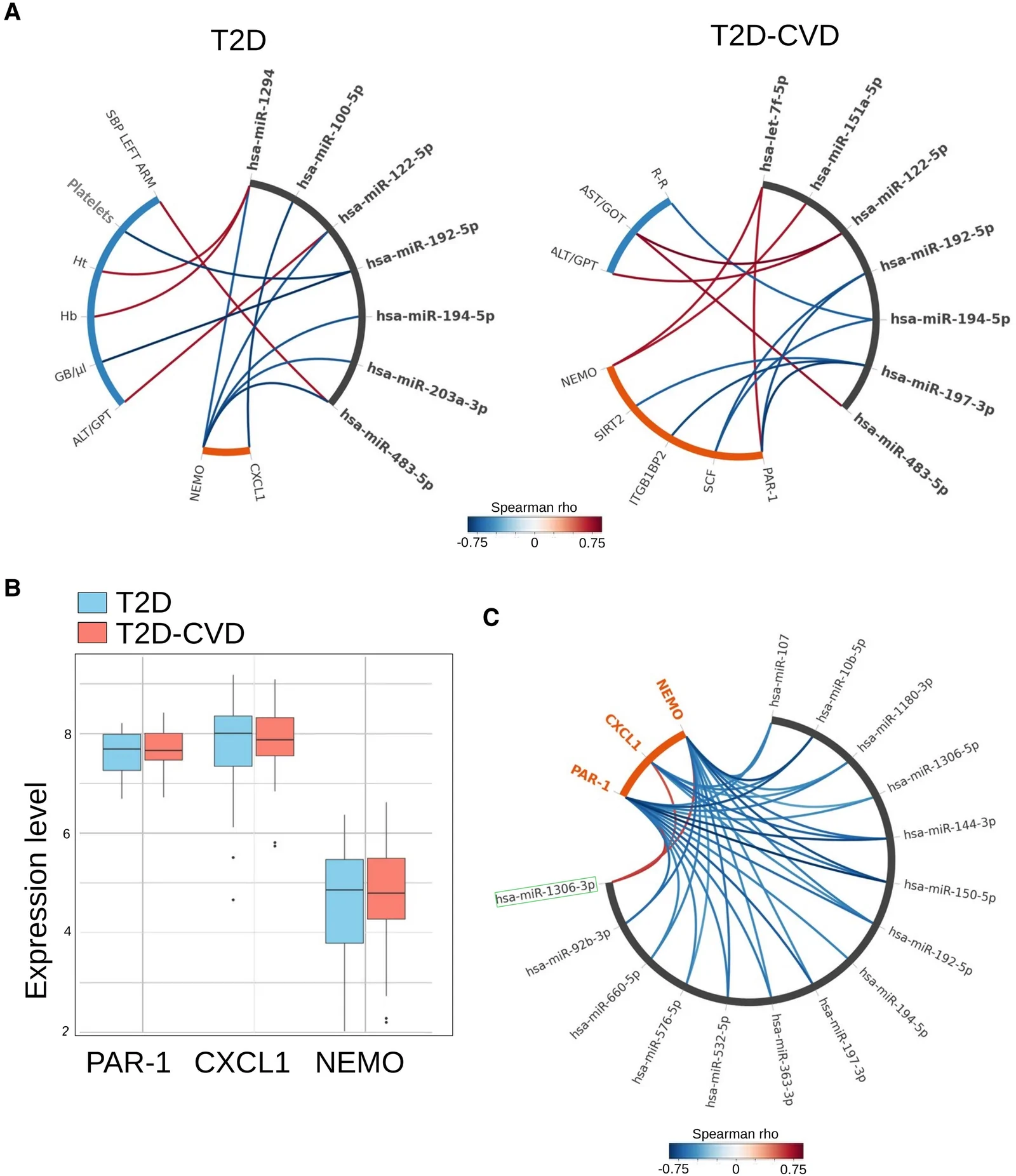
Correlation of miRNAs of interest with circulating immune-inflammatory molecules in T2D-CVD patients. A, Circos diagrams of the statistically significant Spearman correlations between differentially expressed miRNAs and clinical variables/inflammatory factors in T2D (left) and T2D-CVD (right). Nodes are grouped by category around the circle (miRNAs, inflammatory factors, clinical variables); only associations significant after Benjamini-Hochberg correction (FDR-adjusted *P* < .05) are shown. Each arc links a miRNA to the correlated variable; arc shading encodes the magnitude and sign of the Spearman correlation coefficient on a continuous scale (negative to positive), as indicated in the key. B, Circulating levels of the inflammatory factors PAR-1, CXCL1, and NEMO in T2D and T2D-CVD patients, as labelled. Boxes represent the median and interquartile range, whiskers the 1.5× interquartile range, and points the outliers. C, Chord diagram of the significant Spearman correlations between circulating miRNAs of Community 2 and the inflammatory factors PAR-1, CXCL1, and NEMO in T2D-CVD. Arc shading encodes the magnitude and sign of the Spearman correlation coefficient, as in panel A. The miRNA hub miR-1306-3p shows positive correlation with PAR-1, CXCL1, and NEMO, all involved in the Hippo signalling pathway.

A statistically significant negative correlation of 41%, 17%, and 38% community miRNAs with PAR-1, CXCL1, and with NEMO, respectively, was detected (Fig. 6C).

Finally, we observed a statistically significant positive correlation of hub miR-1306-3p with PAR-1, CXCL1, and NEMO (Fig. 6C), including 9 molecules (Supplementary Table S6) (12) involved in inflammation via nuclear factor κB (NF-κB), TGF-β, and p38MAPK signaling pathways, altered ECM and β-cell apoptosis.

Collectively, these data suggest that circulating miRNAs in T2D-CVD patients are tightly associated with immune-inflammatory responses, potentially through the Hippo-signaling pathway and its associated molecules, highlighting their potential as biomarkers for disease progression and identification of novel therapeutic targets in T2D-CVD.

## Discussion

The markedly increased risk of CVD in patients with T2D, together with the global rise in T2D prevalence, highlights the need for precision medicine tools that capture individual CV risk and underlying mechanisms. Recent efforts, such as the Precision Medicine in Diabetes Initiative, have identified several candidate biomarkers for CVD risk prediction in T2D, but circulating miRNAs were not included despite growing evidence of their potential clinical utility (25).

Circulating miRNAs are attractive biomarker candidates because they are stable in plasma, accessible through minimally invasive sampling, and mechanistically linked to insulin resistance, β-cell dysfunction, vascular remodeling, and inflammation. However, prior studies based on conventional DE analyses and small miRNA panels have not yielded reproducible signatures ready for clinical translation, partly because they do not fully account for the multidimensional complexity of immune-metabolic crosstalk (26-28).

In this study, we combined comprehensive profiling of circulating miRNAs with network and community analyses in T2D patients with and without CVD, integrating these data with an extensive characterization of inflammatory cytokines, chemokines, soluble receptors, and atherosclerosis-related molecules. This systems-level approach allowed us to identify a T2D-CVD–specific community of 34 highly interconnected miRNAs that is enriched for inflammatory and cardiometabolic pathways, including mTOR, TGF-β, tumor necrosis factor, and Hippo signaling, pointing to a coordinated regulatory module in diabetic cardiomyopathy.

Differential network analysis further revealed 3 hub miRNAs—let-7a-5p, miR-132-3p, and miR-1306-3p—that are peculiar to T2D-CVD and primarily linked to Hippo signaling, inflammation, vascular remodeling, and cardiac repair. Validation experiments confirmed key subnetworks centered on let-7a-5p (in connection with miR-505-5p) and miR-132-3p (connected with miR-148a-3p, miR-423-5p, and miR-664a-5p), supporting the robustness of these miRNA patterns. Together, these findings suggest a possible association between coordinated dysregulation of specific miRNA communities/hubs and the pathophysiological continuum from T2D to overt CVD, a hypothesis that will require confirmation in larger and longitudinal cohorts.

Literature evidence further supports the functional relevance of the 3 hub miRNAs in T2D-CVD; let-7a-5p mediates anti-inflammatory and antiapoptotic effects via MKP1-dependent regulation of p38 MAPK and JNK pathways (29); miR-132-3p governs pancreatic β-cell survival and vascular smooth muscle cell behavior through distinct signaling cascades (30-32); and miR-1306-3p emerges as a candidate biomarker in postinfarction heart failure (33).

Additionally, we observed significant correlations between the hub miRNA miR-1306-3p and circulating inflammatory mediators PAR-1, CXCL1, and NEMO, all upstream or downstream of the Hippo-signaling pathway. Indeed, PAR-1 acts upstream of CXCL1 and NF-κB pathways in endothelial/immune cells (34) and its correlation with miR-1306 suggests miRNA-mediated modulation of inflammation via PAR-1. CXCL1 is a downstream effector in Hippo-immune crosstalk, partially regulated by MST1 Hippo kinase and expressed in endothelial/immune cells (34) potentially modulated by miRNAs to influence vascular inflammation and remodeling. NEMO is a central hub for NF-κB signaling whose interplay with MST1 links Hippo pathway dynamics to inflammatory feedback (10), thus miRNAs associating with NEMO may influence this regulatory network.

This study provides novel insights by incorporating an miRNA-centric approach to investigate regulatory mechanisms underlying T2D and its CV complications. While previous research has established the critical role of the Hippo-signaling pathway in mediating pancreatic β-cell survival (10, 35) and cardiac regeneration (36) through integrative pathway enrichment analyses of miRNA target genes in our datasets, we observed strong and consistent enrichment of the Hippo-signaling pathway—suggesting that specific miRNAs may serve as upstream regulators of Hippo components. This adds a novel layer to the regulatory circuitry; miRNAs potentially modulate key processes like apoptosis, proliferation, and fibrosis in metabolic tissues and diabetic cardiac injury.

Taken together, our findings are consistent with a role for Hippo signaling in T2D and CV pathophysiology and suggest a previously unappreciated, hypothesis-generating regulatory axis involving miRNAs. This expands the existing framework of molecular crosstalk underpinning metabolic dysfunction and vascular remodeling.

This study has several limitations. First, the overall sample size, and particularly the independent validation cohort (n = 30), is relatively small for high-dimensional circulating miRNA profiling and network-based analyses, which limits statistical power and increases the risk of false-positive findings; indeed, not all differential coexpression edges (eg, those involving hub miR-1306-3p) were confirmed in the validation cohort. Larger, independent, and ideally multicenter cohorts will be required to confirm the generalizability of the proposed miRNA signature. Second, the T2D-CVD group was defined by macrovascular complications encompassing clinically heterogeneous conditions (myocardial infarction, stroke, and peripheral arterial disease); combining these distinct vascular territories and pathophysiological entities may obscure disease-specific miRNA patterns, and our sample size did not allow for adequately powered stratification by CVD subtype. Third, this is a cross-sectional case-control study, which can identify associations between circulating miRNAs and prevalent CVD but cannot determine whether these miRNA alterations precede, contribute to, or simply reflect established CVD; longitudinal studies with incident CV events are needed to evaluate the predictive value of this miRNA network. Fourth, although DE analysis was adjusted for sex and hemolysis, other factors that differed between groups, including smoking history and medication use (statins, insulin, β-blockers, calcium channel blockers), were not incorporated into our models; because such medications are often prescribed as a consequence of CVD status itself, residual confounding by indication cannot be excluded in this observational cohort. Finally, the proposed involvement of Hippo signaling is based on pathway enrichment and network inference using validated miRNA-target databases and correlation analyses; these approaches are hypothesis-generating and do not demonstrate pathway activation or direct miRNA-target regulation. Functional validation in relevant vascular, inflammatory, or metabolic models will be necessary before mechanistic conclusions can be firmly supported.

### Conclusions

We identified a candidate network of circulating miRNAs and cytokine biomarkers associated with T2D-CVD, providing hypothesis-generating insights into potential molecular mechanisms involved in CVD; confirmation in larger, longitudinal, and functionally validated studies will be required before these findings can inform patient stratification or therapeutic strategies.

## Data Availability

Supporting data, including supplementary figures, tables, and supplementary data file 1 (Small RNA seq, Circulating MicroRNAs Normalized Reads Counts_ Median of Ratios- DeSeq2) are available on Mendeley Data (doi:10.17632/srxdmvxvyb.1). All data generated or analyzed during this study are included in this published article or in the data repositories listed in “References” and in this data repository link: https://data.mendeley.com/preview/srxdmvxvyb?a=4e7f6691-7811-4cef-b839-d37c1f719dd1.

## Abbreviations

CVD: cardiovascular disease
ddPCR: droplet digital polymerase chain reaction
DE: differential expression
ECM: extracellular matrix
FC: fold change
FDR: false discovery rate
MAPK: mitogen-activated protein kinase
miRNA: microRNA
mTOR: mechanistic target of rapamycin
NF-κB: (nuclear factor κB)
T2D: type 2 diabetes
T2D-CVD: type 2 diabetes with cardiovascular disease
TGF-β: transforming growth factor-β

## References

[1] Dal Canto E, Ceriello A, Rydén L, et al Diabetes as a cardiovascular risk factor: an overview of global trends of macro and micro vascular complications. Eur J Prev Cardiol. 2019;26(2_suppl):25‐32. 10.1177/2047487319878371 31722562

[2] Laakso M . Cardiovascular disease in type 2 diabetes from population to man to mechanisms: the Kelly West Award Lecture 2008. Diabetes Care. 2010;33(2):442‐449. 10.2337/dc09-0749 PMC2809299 20103560

[3] Einarson TR, Acs A, Ludwig C, Panton UH. Prevalence of cardiovascular disease in type 2 diabetes: a systematic literature review of scientific evidence from across the world in 2007-2017. Cardiovasc Diabetol. 2018;17(1):83. 10.1186/s12933-018-0728-6 PMC5994068 29884191

[4] Nathan DM, Meigs J, Singer DE. The epidemiology of cardiovascular disease in type 2 diabetes mellitus: how sweet it is… or is it? Lancet. 1997;350(suppl 1):SI4‐SI9. 10.1016/s0140-6736(97)90021-0 9250276

[5] Ma CX, Ma XN, Guan CH, Li YD, Mauricio D, Fu SB. Cardiovascular disease in type 2 diabetes mellitus: progress toward personalized management. Cardiovasc Diabetol. 2022;21(1):74. 10.1186/s12933-022-01516-6 PMC9107726 35568946

[6] Shah R, Murthy V, Pacold M, et al Extracellular RNAs are associated with insulin resistance and metabolic phenotypes. Diabetes Care. 2017;40(4):546‐553. 10.2337/dc16-1354 PMC5360281 28183786

[7] Thomou T, Mori MA, Dreyfuss JM, et al Adipose-derived circulating miRNAs regulate gene expression in other tissues. Nature. 2017;542(7642):450‐455. 10.1038/nature21365 PMC5330251 28199304

[8] Silverman EK, Schmidt HHHW, Anastasiadou E, et al Molecular networks in network medicine: development and applications. Wiley Interdiscip Rev Syst Biol Med. 2020;12(6):e1489. 10.1002/wsbm.1489 PMC7955589 32307915

[9] Ardestani A, Lupse B, Maedler K. Hippo signaling: key emerging pathway in cellular and whole-body metabolism. Trends Endocrinol Metab. 2018;29(7):492‐509. 10.1016/j.tem.2018.04.006 29739703

[10] Wang X, Du J, Li H, Cao Z, Cheng Z, Wang Z. The Hippo signaling pathway modulates pancreatic tissue homeostasis. Cell Death Discov. 2025;11(1):343. 10.1038/s41420-025-02636-0 PMC12289952 40707469

[11] Grieco GE, Sebastiani G, Fignani D, et al Protocol to analyze circulating small non-coding RNAs by high-throughput RNA sequencing from human plasma samples. STAR Protoc. 2021;2(3):100606. 10.1016/j.xpro.2021.100606 PMC8219884 34189472

[12] Besharat ZM, Trocchianesi S, Grieco GE, et al 2026. Supplementary data for: Hippo signalling-linked circulating microRNA networks associate with cardiovascular disease in type 2 diabetes. Mendeley Data. doi:10.17632/srxdmvxvyb.1. Version 1. August 24.

[13] Tastsoglou S, Skoufos G, Miliotis M, et al DIANA-miRPath v4.0: expanding target-based miRNA functional analysis in cell-type and tissue contexts. Nucleic Acids Res. 2023;51(W1):W154‐W159. 10.1093/nar/gkad431 PMC10320185 37260078

[14] Kobayashi S, Cox AG, Harvey KF, Hogan BM. Vasculature is getting Hip(po): Hippo signaling in vascular development and disease. Dev Cell. 2023;58(23):2627‐2640. 10.1016/j.devcel.2023.11.002 38052179

[15] Zheng A, Chen Q, Zhang L. The Hippo-YAP pathway in various cardiovascular diseases: focusing on the inflammatory response. Front Immunol. 2022;13:971416. 10.3389/fimmu.2022.971416 PMC9433876 36059522

[16] Mia MM, Singh MK. Emerging roles of the Hippo signaling pathway in modulating immune response and inflammation-driven tissue repair and remodeling. FEBS J. 2022;289(14):4061‐4081. 10.1111/febs.16449 35363945

[17] Lin MOY, Sampath D, Bosykh DA, et al YAP/TAZ drive agrin-matrix metalloproteinase 12-mediated diabetic skin wound healing. J Invest Dermatol. 2025;145(1):155‐170.e2. 10.1016/j.jid.2024.05.005 38810954

[18] Sato T, Akao K, Sato A, Tsujimura T, Mukai S, Sekido Y. Aberrant expression of *NPPB* through YAP1 and TAZ activation in mesothelioma with Hippo pathway gene alterations. Cancer Med. 2023;12(12):13586‐13598. 10.1002/cam4.6056 PMC10315720 37165917

[19] Xu Y, Bai Z, Lan T, Fu C, Cheng P. CD44 and its implication in neoplastic diseases. MedComm (2020). 2024;5(6):e554. 10.1002/mco2.554 PMC11112461 38783892

[20] Malachias MVB, Jhund PS, Claggett BL, et al NT-proBNP by itself predicts death and cardiovascular events in high-risk patients with type 2 diabetes mellitus. J Am Heart Assoc. 2020;9(19):e017462. 10.1161/JAHA.120.017462 PMC7792415 32964800

[21] He J, Bao Q, Yan M, et al The role of Hippo/yes-associated protein signalling in vascular remodelling associated with cardiovascular disease. Br J Pharmacol. 2018;175(8):1354‐1361. 10.1111/bph.13806 PMC5866970 28369744

[22] Moon S, Kim W, Kim S, et al Phosphorylation by NLK inhibits YAP-14-3-3-interactions and induces its nuclear localization. EMBO Rep. 2017;18(1):61‐71. 10.15252/embr.201642683 PMC5210122 27979972

[23] Mo JS, Yu FX, Gong R, Brown JH, Guan KL. Regulation of the Hippo-YAP pathway by protease-activated receptors (PARs). Genes Dev. 2012;26(19):2138‐2143. 10.1101/gad.197582.112 PMC3465735 22972936

[24] Wang S, Zhou L, Ling L, et al The crosstalk between Hippo-YAP pathway and innate immunity. Front Immunol. 2020;11:323. 10.3389/fimmu.2020.00323 PMC7056731 32174922

[25] DeGroat W, Abdelhalim H, Patel K, Mendhe D, Zeeshan S, Ahmed Z. Discovering biomarkers associated and predicting cardiovascular disease with high accuracy using a novel nexus of machine learning techniques for precision medicine. Sci Rep. 2024;14(1):1. 10.1038/s41598-023-50600-8 PMC10762256 38167627

[26] Mahjoob G, Ahmadi Y, Fatima Rajani H, Khanbabaei N, Abolhasani S. Circulating microRNAs as predictive biomarkers of coronary artery diseases in type 2 diabetes patients. J Clin Lab Anal. 2022;36(5):e24380. 10.1002/jcla.24380 PMC9102494 35349731

[27] Szydełko J, Matyjaszek-Matuszek B. MicroRNAs as biomarkers for coronary artery disease related to type 2 diabetes mellitus—from pathogenesis to potential clinical application. Int J Mol Sci. 2022;24(1):616. 10.3390/ijms24010616 PMC9820734 36614057

[28] Saini VM, Liu KR, Surve AS, Gupta S, Gupta A. MicroRNAs as biomarkers for monitoring cardiovascular changes in type II diabetes mellitus (T2DM) and exercise. J Diabetes Metab Disord. 2022;21(2):1819‐1832. 10.1007/s40200-022-01066-4 PMC9261151 35818628

[29] Wang ZK, Liu FF, Wang Y, Jiang XM, Yu XF. Let-7a gene knockdown protects against cerebral ischemia/reperfusion injury. Neural Regen Res. 2016;11(2):262‐269. 10.4103/1673-5374.177734 PMC4810990 27073379

[30] Mziaut H, Henniger G, Ganss K, et al MiR-132 controls pancreatic beta cell proliferation and survival through Pten/Akt/Foxo3 signaling. Mol Metab. 2020;31:150‐162. 10.1016/j.molmet.2019.11.012 PMC6928290 31918917

[31] Xu Q, Liang Y, Liu X, et al miR-132 inhibits high glucose-induced vascular smooth muscle cell proliferation and migration by targeting E2F5. Mol Med Rep. 2019;20(2):2012‐2020. 10.3892/mmr.2019.10380 31257477

[32] Zeller T, Keller T, Ojeda F, et al Assessment of microRNAs in patients with unstable angina pectoris. Eur Heart J. 2014;35(31):2106‐2114. 10.1093/eurheartj/ehu151 24727883

[33] Liang J, Bai S, Su L, et al A subset of circulating microRNAs is expressed differently in patients with myocardial infarction. Mol Med Rep. 2015;12(1):243‐247. 10.3892/mmr.2015.3422 25738515

[34] Antoniak S, Tatsumi K, Schmedes CM, et al PAR1 regulation of CXCL1 expression and neutrophil recruitment to the lung in mice infected with influenza A virus. J Thromb Haemost. 2021;19(4):1103‐1111. 10.1111/jth.15221 PMC8048419 33346953

[35] Ardestani A, Paroni F, Azizi Z, et al MST1 is a key regulator of beta cell apoptosis and dysfunction in diabetes. Nat Med. 2014;20(4):385‐397. 10.1038/nm.3482 PMC3981675 24633305

[36] Zhou Q, Li L, Zhao B, Guan KL. The Hippo pathway in heart development, regeneration, and diseases. Circ Res. 2015;116(8):1431‐1447. 10.1161/CIRCRESAHA.116.303311 PMC4394208 25858067

